# Healthcare Professionals’ Breastfeeding Attitudes and Hospital Practices During Delivery and in Neonatal Intensive Care Units: Pre and Post Implementing the Baby-Friendly Hospital Initiative

**DOI:** 10.1177/08903344211058373

**Published:** 2021-11-28

**Authors:** Heli Mäkelä, Anna Axelin, Terhi Kolari, Tuula Kuivalainen, Hannakaisa Niela-Vilén

**Affiliations:** 1 Department of Nursing Science, University of Turku, Turku, Finland; 2 Satakunta Hospital District, Satasairaala, Pori, Finland; 3 University on Turku, Department of Biostatistics, University of Turku, Turku, Finland

**Keywords:** Baby-Friendly Hospital Initiative, Baby-Friendly Hospital Initiative for neonatal wards, breastfeeding, breastfeeding attitude, breastfeeding practices, healthcare professionals, Neo-BFHI, neonatology, neonatal intensive care unit (NICU), postpartum care, quasi-experimental study

## Abstract

**Background:**

The Baby-Friendly Hospital Initiative represents a global effort to support breastfeeding. Commitment to this program has been associated with the longer duration and exclusivity of breastfeeding and improvements in hospital practices. Further, healthcare professionals’ breastfeeding attitudes have been associated with the ability to provide professional support for breastfeeding.

**Research Aims:**

To determine healthcare professionals’ breastfeeding attitudes and hospital practices before and after the implementation of the Baby-Friendly Hospital Initiative.

**Methods:**

Using a quasi-experimental pretest–posttest study design, healthcare professionals (*N* = 131) from the single hospital labor and delivery, maternity care, and neonatal intensive care were recruited before and after the Baby-Friendly Hospital Initiative intervention during 2017 and 2019. Breastfeeding attitudes with the validated *Breastfeeding Attitude Questionnaire*, breastfeeding-related hospital practices, and background characteristics were collected.

**Results:**

The healthcare professionals’ breastfeeding attitude scores increased significantly after the implementation of the Baby-Friendly Hospital Initiative, difference = 0.16, (95% CI [0.13, 0.19]) and became breastfeeding favorable among all professional groups in each study unit. Positive changes in breastfeeding-supportive hospital practices were achieved. The infants had significantly more frequent immediate and uninterrupted skin-to-skin contact with their mothers. The rate of early breastfeeding, as well as the number of exclusively breastfed infants, increased.

**Conclusions:**

After the Baby-Friendly Hospital Initiative and Baby-Friendly Hospital Initiative for neonatal wards (Neo-BFHI) interventions were concluded, we found significant improvements in the breastfeeding attitudes of healthcare professionals and in breastfeeding-related care practices.

This RCT was registered (0307-0041) with ClinicalTrials.gov on 03/03/2017.

## Tiivistelmä

**Tausta:**

Vauvamyönteisyysohjelma on Maailman terveysjärjestö WHO:n ja lastenjärjestö UNICEFin vuonna 1991 käynnistämä hanke imetyksen suojelemiseksi ja edistämiseksi. Ohjelmaan sitoutumisen on todettu lisäävän imetystä, pidentävän imetyksen kestoa ja parantavan imetystä tukevia hoitokäytäntöjä. Myös henkilökunnan imetysasenne on yhteydessä imetysohjaukseen ja hoitokäytänteisiin.

**Tarkoitus:**

Tutkimuksen tarkoituksena oli arvioida Vauvamyönteisyysohjelman vaikutusta henkilökunnan imetysasenteisiin sekä imetystä edistäviin ja tukeviin hoitokäytänteisiin.

**Menetelmä:**

Tutkimus toteutettiin kvasikokeellisella ennen-jälkeen asetelmalla. Ensimmäinen aineisto kerättiin keväällä 2017 ennen Vauvamyönteisyysohjelman implementointia ja toinen aineisto kerättiin samoilta vastaajilta keväällä 2019 Vauvamyönteisyys-sertifikaatin myöntämisen jälkeen. Aineisto kerättiin Ruotsissa kehitetyllä imetysasennemittarilla synnytysyksikön sekä vastasyntyneiden teho- ja tarkkailuosaston koko henkilökunnalta (*N* = 131). Asennekyselyn lisäksi tarkasteltiin yksiköiden imetystä tukevia hoitokäytäntöjä ja henkilökunnan taustatietoja.

**Tulokset:**

Henkilökunnan imetysasennepistemäärä nousi Vauvamyönteisyys-sertifikaatin myöntämisen jälkeen erotus = 0.16, 95% luottamusväli [0.13, 0.19] ja asenne muuttui imetysmyönteisempään suuntaan kaikissa ammattiryhmissä sekä kaikissa yksiköissä. Sairaalan tilastojen perusteella imetystä tukevat hoitokäytännöt paranivat. Varhainen ja katkeamaton ihokontakti lisääntyi merkittävästi. Ensi-imetys toteutui useammin ja täysimetettyjen vauvojen määrä lisääntyi Vauvamyönteisyysohjelman implementoinnin jälkeen.

**Johtopäätökset:**

Vauvamyönteisyysohjelma paransi henkilökunnan imetysasennetta ja imetystä tukevia hoitokäytäntöjä imetysmyönteisemmäksi sekä synnytysyksikössä että vastasyntyneiden teho- ja tarkkailuosastolla.

Tämä tutkimus on rekisteröity (0307-0041) ClinicalTrials.gov -rekisteriin 03/03/2017.

Back translation by: Riikka Ikonen, RN, PhD

## Key Messages

- The influence of the Baby-Friendly Hospital Initiative and the Baby-Friendly Hospital Initiative for neonatal wards designations on healthcare professionals’ attitude has not been systematically studied, and evidence of the influence of the Baby-Friendly Hospital Initiative for neonatal wards on breastfeeding outcomes is lacking.
- In our study, healthcare professionals’ attitude toward breastfeeding became more favorable after the Baby-Friendly Hospital Initiative and the Baby-Friendly Hospital Initiative for neonatal wards designations.

## Background

The Baby-Friendly Hospital Initiative (BFHI) is a global effort to implement practices that protect, promote, and support breastfeeding, established in 1991 (World Health Organization [WHO], 2018). A version of the BFHI for neonatal intensive care units (NICUs), the Neo-BFHI, was established in 2015 to meet the special needs of preterm and low-birthweight infants and their mothers (Nyqvist et al., 2012; 2013; 2015). Nearly all countries have implemented some of the Baby-Friendly principles, but many countries still struggle to implement the whole program (WHO, 2018). Neo-BFHI assessments are recommended to be conducted at the same time with BFHI, but they are separate designations (Nyqvist et al., 2015). Continuous work is needed after the implementation process, because BFHI standards may decline rapidly after the designation (Zakarija-Grkovic et al., 2018).

Commitment to the BFHI program, particularly the Ten Steps to Successful Breastfeeding, has increased breastfeeding rates and been associated with longer duration and exclusivity of breastfeeding (Howe-Heyman & Lutenbacher, 2016; Munn et al., 2016; Pérez-Escamilla et al., 2016). Full implementation of the BFHI program has resulted in improvements in hospital practices, for example, early breastfeeding initiation, rooming-in, skin-to-skin contact (SSC), and giving no artificial teats (Agbozo et al., 2020; Alonso-Díaz et al., 2016; Araújo et al., 2019; Gomez-Pomar & Blubaugh, 2018; Zakarija-Grković et al., 2018). Implementation of the original BFHI has been beneficial in neonatal ward policies and practices as well, but the influences of the Neo-BFHI on breastfeeding outcomes is lacking (Maastrup et al., 2019).

Healthcare professionals’ (HCPs) attitudes toward breastfeeding have been associated with the quality of care and with HCPs’ ability to provide professional support for breastfeeding (Ekström & Thorstensson, 2015; Sigman-Grant & Kim, 2016). Individual and tailored support for mothers with healthy term infants has increased the duration and exclusivity of breastfeeding (McFadden et al., 2017) and decreased mothers’ breastfeeding challenges (Ekström & Thorstensson, 2015). For breastfeeding support in NICUs, HCPs’ ability to create a good relationship with the mother (Gianni et al., 2018) and a positive attitude about breastfeeding are essential (Shattnawi, 2017).

Previously, researchers have shown that education and training about breastfeeding improved HCPs’ attitudes toward breastfeeding, as well as the consistency of their advice (Rosen-Carole et al., 2016; Shattnawi, 2017; Yang et al., 2018), and professional support they were able to provide for breastfeeding mothers (Ekström & Thorstensson, 2015). Less professional experience and younger ages have been associated with a positive breastfeeding attitude and an increased awareness of the importance of breastfeeding education (Vizzari et al., 2020). Previous personal breastfeeding experiences also have been found to positively influence HCPs’ attitude toward breastfeeding (Yang et al., 2018).

HCPs have a crucial role in supporting breastfeeding in hospitals and their attitude and knowledge to provide high-quality, individual breastfeeding support for breastfeeding are essential for its success (Shattnawi, 2017). The influence of the BFHI and Neo-BFHI on HCPs’ attitudes needs to be understood better, and whether they affect hospital practices should be examined. The aim of this study was to assess HCPs’ breastfeeding attitudes and breastfeeding-related hospital practices before and after the implementation of the BFHI and Neo-BFHI.

## Methods

### Research Design

A quasi-experimental pretest–posttest study design was used (Siedlecki, 2020) to compare HCPs breastfeeding attitudes and hospital practices before and after the BFHI and Neo-BFHI implementation. The study was conducted in accordance with the Helsinki Declaration of 2013 (World Medical Association, 2013). The study protocol received a favorable statement by the Ethics Committee at the University of Turku (statement 18/2017) and was approved by the hospital administration.

### Setting and Relevant Context

The study was conducted in a public, Level II hospital in Western Finland with approximately 1,700 childbirths annually and approximately 400 yearly admissions in the NICU. The average hospital stay was 2 days after a vaginal birth and 3 days after a cesarean section. Mothers were encouraged to “room-in” with their infants 24/7 in the NICU. The study units were 1) a maternity outpatient clinic; 2) a labor and delivery unit; 3) a maternity unit including both prenatal and postnatal patients; and 4) a NICU.

### Sample

Our target population comprised the HCPs (*N* = 131) working at the study units during the spring of 2017. The total population sampling method was used, and all HCPs were eligible and invited to participate. The nursing professionals were nurses (*n* = 34, 26%) and midwives (*n* = 71, 54%); the rest of the HCPs were pediatricians (*n* = 11, 8%) and obstetricians (*n* = 15, 11%). The researcher informed HCPs of the opportunity to participate in the study verbally at a staff meeting. A description of the study was sent by email after the meeting.

**Figure 1.**
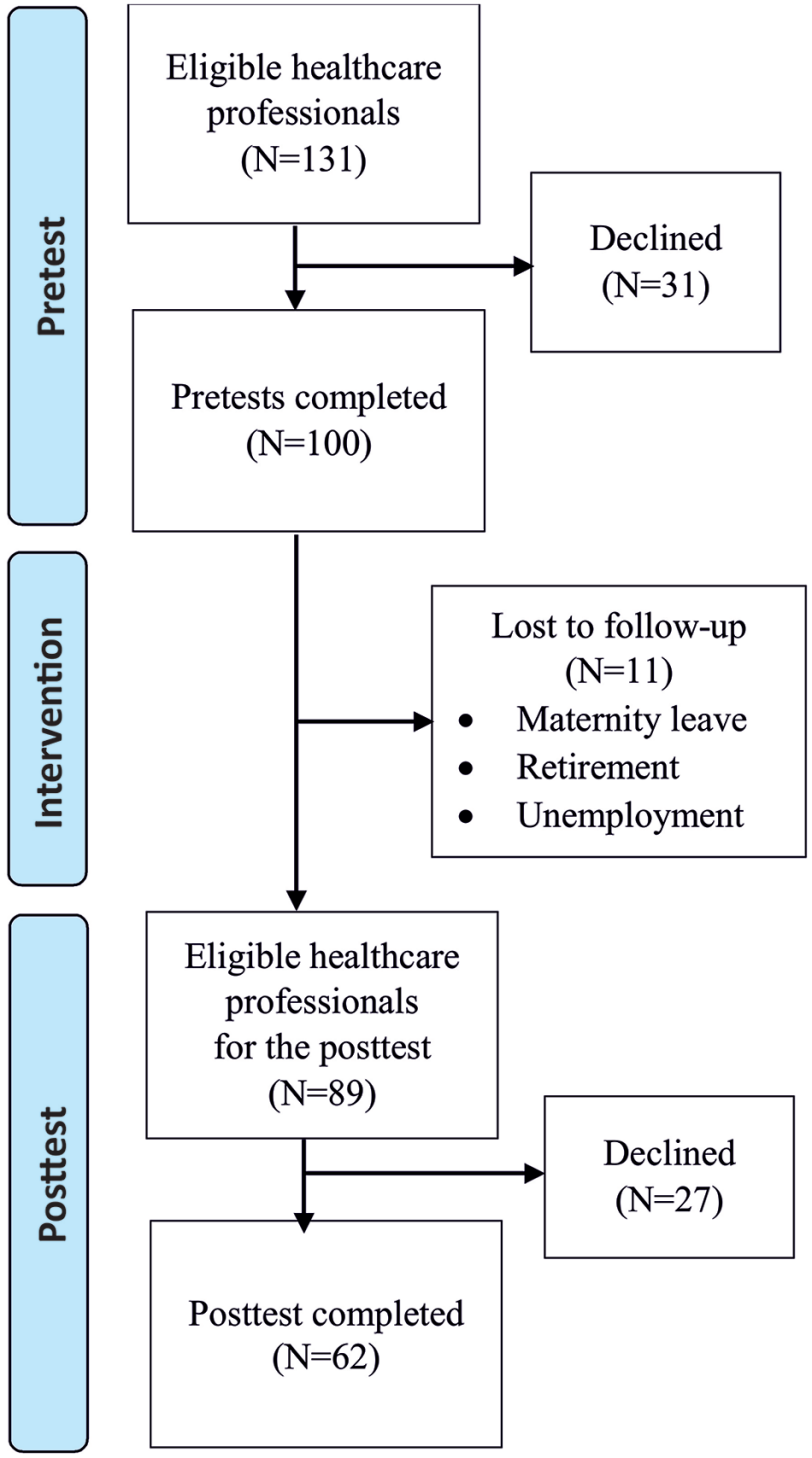
Flowchart of the Data Collection.

Of all eligible candidates (*N* = 131), 76% (*n* = 100) participated in the pretest. The posttest sample included *n* = 62 (62%) participants (Figure 1).

### Measurements

HCPs’ attitudes were measured using the previously developed Breastfeeding Attitude Questionnaire (Ekström et al., 2005). The questionnaire includes 47 items with a 4-point Likert-scale, the response options ranging from “disagree completely" to “agree completely.” The mean score of the instrument ranges from 1–4, with higher scores indicating a more favorable attitude toward breastfeeding. Furthermore, to represent different types of the breastfeeding attitude, the items are distributed among four factors. The “regulating factor” (10 items) focuses on HCPs’ orientation on mothers’ breastfeeding management and contains statements about advising and scheduling feeding. The “facilitating factor” (9 items) focused on HCPs’ support for mothers managing their own breastfeeding, containing statements about evidence-based practices and support for breastfeeding. The “disempowering factor” (7 items) focused on giving professional advice, without regard for the needs of the mother receiving counseling. The “breastfeeding-antipathy factor” (9 items) focused on HCPs’ insufficient basic breastfeeding knowledge and hostile reactions to breastfeeding. The originally reported Cronbach’s alpha reliability coefficient was 0.51 for the instrument, and factor scores range from 0.29 to 0.80 (Ekström et al., 2005). Despite the reliability limitations, this tool was selected because it directly measures HCPs’ attitudes toward breastfeeding.

Demographic characteristics (age, profession, work experience, working unit, and personal breastfeeding experiences) were collected with a self-report questionnaire developed for this study. The questionnaire also included dichotomized questions (yes/no) about previous breastfeeding education and a need for more education.

Statistics about breastfeeding-related hospital practices (the timing of first breastfeeding after birth and dichotomized values (yes/no) about exclusive breastfeeding after birth and at discharge, immediate and uninterrupted SSC, the use of a medically indicated supplementation, and the use of a pacifier and nipple shield) were collected for all admissions with the information form developed for regular monitoring of the hospital practices.

### The Intervention: Implementation of the BFHI and the Neo-BFHI

**Figure 2.**
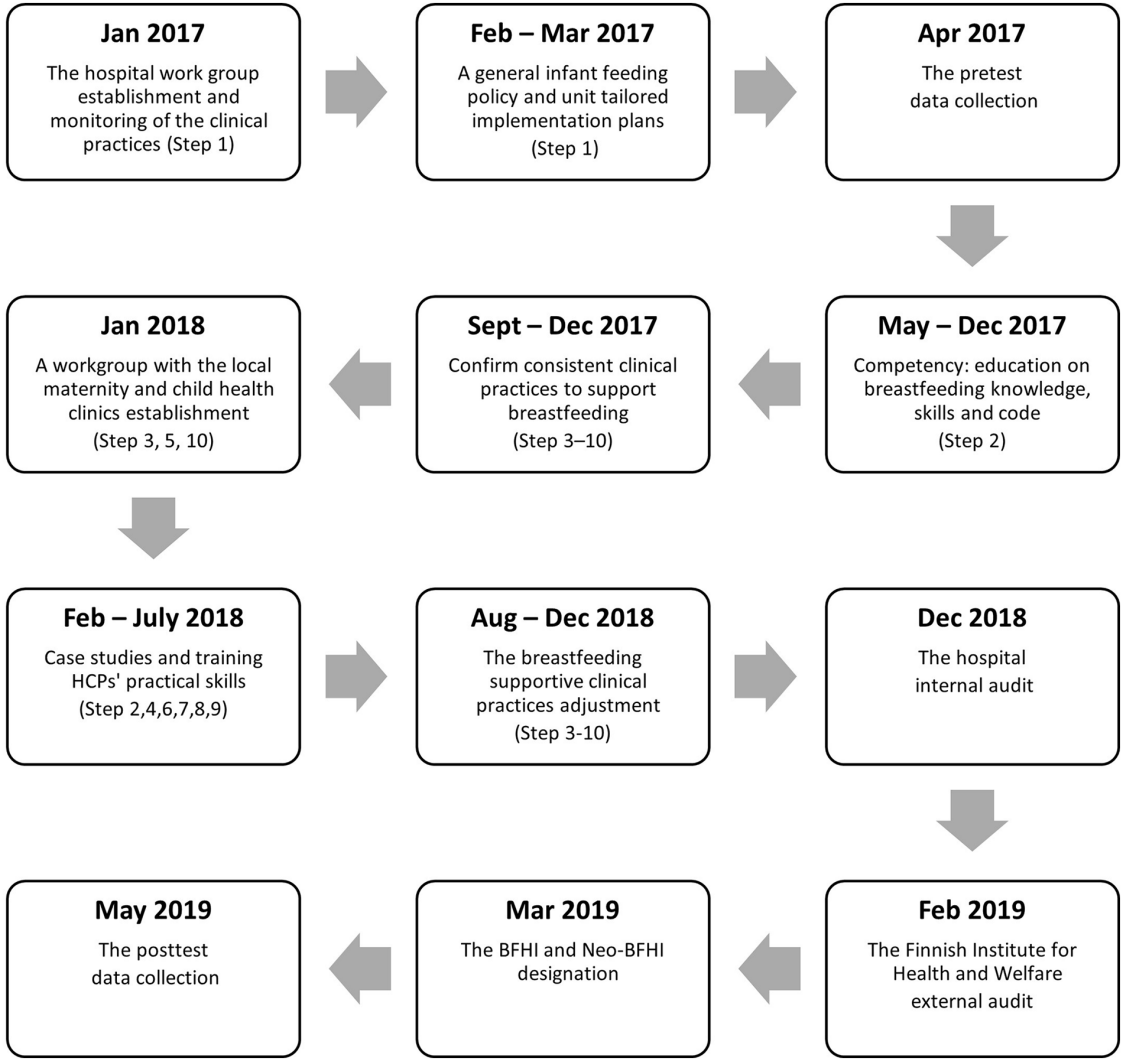
Timeline of the BFHI and Neo-BFHI Implementation Process and the Data Collection.

The planning and implementation of the BFHI and Neo-BFHI interventions at the study hospital were coordinated by a work group of HCPs from each unit. Implementation protocols and unit-tailored care commitments were designed according to the Ten Steps to Successful Breastfeeding (WHO, 2018) and the Finnish national guidelines promoting and supporting breastfeeding (Hakulinen et al., 2017). The implementation planning began in January 2017, and the process continued for 26 months (Figure 2).

During the interventions, all nursing professionals received education by attending the WHO/United Nations Children's Fund (UNICEF) 20-hr breastfeeding counsellor course. Physicians and nursing professionals who had previously attended the course (74%) attended a 6-hr update course. All other staff members, for example, cleaning staff, attended a 2-hr briefing. Other efforts to strengthen learning were individual task cards for members of each group of professionals, weekly prep cards with a special focus, case studies, and a tool to measure whether the intended guidance was reaching the target groups, the mothers, and families. Regular monitoring of the hospital care practices was established to see the progression and to indicate full implementation of all Ten Steps.

An external audit was performed by experts appointed by the Finnish Institute of Health and Welfare in February 2019. Both the BFHI and Neo-BFHI designations were granted in March 2019.

### Data Collection

The pretest data were collected in April 2017, before the implementation of BFHI and Neo-BFHI. The researcher distributed the pretest questionnaires to all HCPs (*N* = 131) working in the study units. The questionnaires were completed individually during working hours and returned in closed envelopes to the locked case in the unit. Completing the questionnaire was considered consent to the study.

The posttest data were collected in May 2019, within 2 months following the BFHI and Neo-BFHI designations. The researcher responsible for the study delivered a posttest questionnaire to participants who had completed the pretest questionnaire.

Hospital practices from 2017, 2018, and 2019 in the labor and delivery and maternity units and years 2018 and 2019 in the NICU were collected for all admissions. Monitoring of the practices began at the NICU in 2018.

### Data Analysis

Responses to the questionnaire items were numerically coded, and the items with negative loadings were reverse coded. Total breastfeeding attitude scores and the scores for each factor were calculated as a mean of items. Data from all participants from each group were used in the analyses if participant responded to 70% of the items.

Descriptive statistics were used to analyze demographic data. The association of data collection timepoint (pretest and posttest) and background characteristics (group) on the scores were analyzed using a hierarchical linear mixed model with repeated measures, including one within-factor variable (time), one between-factor variable (group), and their interaction (time*group). An unstructured covariance structure was used for time and Kenward-Roger correction for degrees of freedom was conducted. After univariate analysis, all significant factors (timepoint, profession, profession * timepoint, hospital unit * timepoint, previous education in breastfeeding * timepoint) were added to the multivariate model. After backwards elimination, timepoint, the interaction of profession and timepoint, and the interaction of hospital unit and timepoint were included in the final model. In the case of multiple comparisons, Bonferroni-adjusted *p-*values were used. The differences of the proportions with 95% CIs were calculated for the hospital practices. All tests were performed as two-sided, with significance level set at 0.05. The analyses were carried out using the SAS System, Version 9.4 for Windows (SAS Institute Inc., Cary, NC, US).

## Results

### Characteristics of the Participants

The pretest participants’ mean age was 43 years (*SD* = 10.9). Work experience varied from less than 1 year to 40 years, while the mean was 16 years (*SD* = 10.9). Before the BFHI and Neo-BHI implementation, 79% (*n* = 79) of participants had some previous education on breastfeeding, and 74% (*n* = 74) of participants had completed the WHO 20-hr breastfeeding counsellor course. More than half (61%, *n* = 61) of the pretest participants reported a need for further education (Table 1).

**Table 1.** Characteristics of the Participants.

Characteristic	Pretest *n* = 100 *n* (%)	Posttest *n* = 62 *n* (%)
Profession		
Midwife	54 (54.0)	40 (64.5)
Nurse	28 (28.0)	12 (19.4)
Physician	11 (11.0)	5 (8.1)
Other	7 (7.0)	5 (8.1)
Working unit		
Maternity OP clinic	14 (14.0)	11 (17.7)
Delivery unit	21 (21.0)	16 (25.8)
Maternity unit	43 (43.0)	26 (41.9)
NICU	22 (22.0)	9 (14.5)
Previous education in BF		
All	79 (79.0)	
Midwife	53 (98.0)	
Nurse	22 (79.0)	
Physician	0 (0)	
Breastfed as an infant	80 (80.0)	
Has own children	80 (80.0)	
Own children were BF	82 (97.6)	

*Note.* Missing values: Breastfed as an infant = 8; has own children = 6; own children were breastfed = 16. OP = outpatient; NICU = neonatal intensive care unit; BF = breastfeeding/breastfed.

Participants lost between the data collection timepoints (*n* = 11, 11%) were younger (*M* = 34 years, *SD* = 10.9) compared with the posttest data participants (*M* = 44 years, *SD* = 10.4). The participants who dropped out (*n* = 27, 30%) were more often nurses (Fisher exact test *p* = .013) with no previous education on breastfeeding (Fisher exact test *p* = .004) compared with the posttest data participants. The breastfeeding attitude score of participants who dropped out at the posttest data collection point did not differ (Fisher exact test *p* = .453) from the participants who participated to the posttest study.

### Breastfeeding Attitudes

The HCPs’ breastfeeding attitudes improved significantly (*M* difference = 0.16, 95% CI [0.13, 0.19]) after the BFHI and Neo-BFHI designations. Categorized by profession, the midwives’ (*M* difference = 0.15, 95% CI [0.08, 0.22]) and physicians’ (*M* difference = 0.40, 95% CI [0.18, 0.62]) attitudes toward breastfeeding improved to a statistically significant extent. Nurses (difference = 0.12, 95% CI [-0.01, 0.24]) and other professionals’ (difference = 0.12, 95% CI [-0.09, 0.33]) attitudes also became more favorable, but the increase was not statistically significant. Categorized by units, improvements in breastfeeding attitudes were statistically significant in each study unit. (Table 2.)

**Table 2.** Breastfeeding Attitude Scale (*M*) Pre and Post Implementation of the BFHI and the Neo-BFHI.

Breastfeeding Attitude	Pre BFHI		Post BFHI		*AD [95 % CI]*
	*n*	*M [95 % CI]*	*n*	*M [95 % CI]*	
All	100	3.09 [2.06, 3.13]	62	3.25 [3.21, 3.29]	0.16 [0.13, 0.19]
Profession					
Midwife	54	3.13 [3.08, 3.17]	40	3.28 [3.22, 3.33]	0.15 [0.08, 0.22]
Nurse	28	3.06 [3.00, 3.13]	12	3.18 [3.07, 3.28]	0.40 [0.18, 0.62]
Physician	11	2.88 [2.75, 3.01]	5	3.28 [3.10, 3.47]	0.40 [0.18, 0.62]
Other	7	3.03 [2.89, 3.17]	5	3.15 [3.00, 3.31]	0.12 [-0.09, 0.33]
Hospital unit					
Maternity OP clinic	14	3.03 [2.94, 3.13]	11	3.27 [3.17, 3.37]	0.24 [0.17, 0.30]
Labor and delivery	21	3.07 [2.99, 3.14]	16	3.26 [3.18, 3.33]	0.19 [0.13, 0.24]
Maternity unit	43	3.13 [3.08, 3.19]	26	3.24 [3.18, 3.30]	0.11 [0.06, 0.15]
NICU	22	3.05 [2.98, 3.13]	9	3.23 [3.13, 3.33]	0.18 [0.09, 0.27]

*Note.* Absolute difference was calculated between the breastfeeding attitude post BFHI and pre BFHI. OP = outpatient; NICU = neonatal intensive care unit; AD = absolute difference.

Regarding the breastfeeding attitude scale’s factors, scores increased significantly for the facilitating (*M* difference = 0.24, 95% CI [0.14, 0.46]) and disempowering (*M* difference = 0.26, 95% CI [0.09, 0.42]) factors and decreased for the breastfeeding antipathy (*M* difference = -0.14, 95% CI [-0.32, -0.08]) factor. The decrease in attitude scores for the regulating factor was not statistically significant (*M* difference = -0.03, 95% CI [-0.10, 0.13]; Table 3).

**Table 3.** Mean Scores and 95% Confidence Intervals for the Attitude Factors Scores Before and After the Implementation of the BFHI and the Neo-BFHI.

**Attitude Factor**	**Regulating**			**Facilitating**			**Disempowering**			**Breastfeeding** antipathy ^a^		
	Pre *M*	Post *M*	Difference *[95% CI]*	Pre *M*	Post *M*	Difference *[95% CI]*	Pre *M*	Post *M*	Difference *[95% CI]*	Pre *M*	Post *M*	Difference *[95% CI]*
**All**	2.65	2.62	−0.03 [−0.10, 0.13]	3.44	3.68	0.24 [0.14, 0.46]	2.84	3.10	0.26 [0.09, 0.42]	1.55	1.41	−0.14 [−0.32, −0.08]
**Profession**												
**Midwife**	2.62	2.58	−0.04 [−0.15, 0.07]	3.52	3.71	0.19 [0.06, 0.33]	2.74	3.06	0.32 [0.18, 0.46]	1.49	1.38	−0.11 [−0.22, −0.01]
**Nurse**	2.63	2.55	−0.08 [−0.26, 0.10]	3.37	3.65	0.28 [0.05, 0.52]	3.04	3.14	0.10 [−0.14, 0.35]	1.62	1.44	−0.18 [−0.36, −0.01]
**Physician**	2.92	2.90	−0.02 [−0.30, 0.26]	3.04	3.68	0.64 [0.26, 1.02]	2.92	3.32	0.40 [−0.01, 0.81]	1.78	1.42	−0.36 [−0.65, −0.07]
**Hospital unit**												
**Maternity OPc**	2.69	2.67	−0.02 [−0.23, 0.20]	3.45	3.74	0.29 [0.01, 0.56]	2.99	3.17	0.18 [−0.11, 0.47]	1.51	1.33	−0.18 [−0.39, 0.03]
**Labor & delivery**	2.64	2.56	−0.08 [−0.26, 0.15]	3.38	3.59	0.21 [−0.01, 0.44]	2.66	2.99	0.33 [0.09, 0.56]	1.62	1.41	−0.21 [−0.38, −0.03]
**Maternity unit**	2.62	2.62	0.0 [−0.12, 0.14]	3.53	3.72	0.19 [0.02, 0.37]	2.84	3.08	0.24 [0.07, 0.42]	1.52	1.41	−0.11 [−0.24, 0.02]
**NICU**	2.74	2.69	−0.05 [−0.27, 0.17]	3.30	3.63	0.33 [0.16, 0.70]	2.93	3.18	0.25 [−0.04, 0.54]	1.60	1.38	−0.22 [−0.43, −0.01]

*Note.* Breastfeeding Attitude Factors score ranged from 1–4 with higher scores indicating more positive attitudes on Regulating, Facilitating, and Disempowering. Pre = 2017 before the BFHI and Neo-BFHI implementation and Post = 2019 after the BFHI and Neo-BFHI implementation. OPc = outpatient clinic; NICU = neonatal intensive care unit.

a On breastfeeding antipathy lower scores indicated more positive attitudes.

### Variables Associated With the Change in Breastfeeding Attitudes

Participants’ profession explained the increase in breastfeeding attitudes to a statistically significant extent. Before the BFHI and Neo-BFHI designations, physicians had lower breastfeeding attitudes (*M* = 2.88, 95% CI [2.75, 3.01]) compared with midwives (*M* = 3.13, 95% CI [3.08, 3.17]) or nurses (*M* = 3.06, 95% CI [3.00, 3.13]), whereas posttest data showed no statistically significant differences between professions.

Previous education about breastfeeding was also significantly associated with breastfeeding attitudes. Before the BFHI designation, HCPs with no previous education on breastfeeding had lower attitudes (*M* = 2.97, 95% CI [2.89, 3.05]) than did professionals who had some previous education on breastfeeding (*M* = 3.11, 95% CI [3.08, 3.15]). During the intervention, all professionals received education, and attitudes improved for both participants with previous education (*M* = 3.11, 95% CI [3.08, 3.15] to *M* = 3.26, 95% CI [3.21, 3.30]) and participants with no previous education (*M* = 2.97, 95% CI [2.89, 3.05] to *M* = 3.26, 95% CI [3.14, 3.37]).

The participants’ age and work experience were not statistically significantly associated with breastfeeding attitudes. Neither the mean scores on breastfeeding attitude nor any of the four factors differed significantly based on whether the participant had personal experiences with breastfeeding—for example, had been breastfed as an infant, had children, or had breastfed/had a partner who had breastfed their children.

In the final multivariate model, timepoint (*M* = 3.01, 95% CI [2.96, 3.06] to *M* = 3.23 95% CI [3.17, 3.29]) and the interactions of timepoint and profession as well as timepoint and unit explained the increase in breastfeeding attitude scores as statistically significant (Table 4).

**Table 4.** Variables Associated With Healthcare Professionals Breastfeeding Attitude in a Final Multivariate Model.

Variable	Pretest	Posttest	*F*_df_	*p*
	*M* [95 % CI]	*M* [95 % CI]		
**Time point**	3.01 [2.96, 3.06]	3.23 [3.17, 3.29]	85.76 _1_	< 0.001
Profession * Time point			3.31 _6_	0.006
**Midwife**	3.12 [3.08, 3.18]	3.29 [3.23, 3.35]		
**Nurse**	3.03 [2.97, 3.10]	3.19 [3.09, 3.28]		
**Physician**	2.86 [2.73, 2.99]	3.22 [3.06, 3.38]		
**Other**	3.02 [2.89, 3.16]	3.22 [3.06, 3.37]		
Hospital Unit * Time point			3.15 _6_	0.008
**Maternity OP clinic**	2.96 [2.87, 3.06]	3.26 [3.15, 3.37]		
**Labor and delivery**	2.96 [2.86, 3.05]	3.20 [3.09, 3.31]		
**Maternity unit**	3.06 [2.99, 3.12]	3.21 [3.13, 3.29]		
**NICU**	3.06 [2.98, 3.14]	3.25 [3.14, 3.36]		

*Note.* All significant variables were added to the multivariate model and, from this model, nonsignificant variables were gradually omitted. The final multivariate model included time point, the interaction of profession and time point and the interaction of hospital unit and time point.

OP = outpatient; NICU = neonatal intensive care unit.

### Hospital Practices

Based on hospital statistics, after the implementation of the BFHI and Neo-BFHI interventions, the hospital practices better promoted and supported breastfeeding after the interventions in the labor and delivery and maternity units as well as the NICU (Table 5). The infants had significantly more frequent immediate and uninterrupted SSC with their mothers after vaginal and caesarian deliveries, and the rate of early breastfeeding increased. One important reason for this was a new procedure in which infants who needed intensive monitoring immediately after birth were returned for SSC with their mothers as soon as possible. The number of infants who were exclusively breastfed and did not receive any supplementary nutrition (donated human milk or formula) during hospital stays increased in both units. The most common reasons for medically indicated supplementation were weight loss, low blood sugar, hyperbilirubinemia, and preterm birth.

**Table 5.** The Changes in Hospital Practices Before and After the Implementation of the BFHI and the Neo-BFHI.

Hospital Practice	Labor and Delivery/Maternity			NICU		
	2017 *n* (%)	2019 *n* (%)	*AD* [*95 % CI*]	2018 *n* (%)	2019 *n* (%)	*AD* [*95 % CI*]
Infants in unit / year	1538	1301		201	177	
Early SSC after birth	1275 (83)	1232 (95)	12 [9.9, 14.1]	125 (62)	120 (68)	6 [-0.8, 12.8]
Breastfeeding <1h after birth (NICU <2h)	1415 (92)	1275 (98)	6 [4.5, 7.5]	121 (60)	177 (66)	6 [-0.8, 12.8]
Rooming in	1307 (85)	1236 (95)	10 [8.0, 12.0]	NA	NA	NA
EB at breast after birth ^a^	615 (40)	696 (53)	13 [-1.3, 5.3]	12 (6)	14 (8)	2 [-1.6, 5.6]
Medically indicated supplementation ^a^	615 (40)	398 (31)	−9 [−12.3, −5.7]	174 (87)	135 (76)	−11[−16.5, −5.5]
Supplementation, but EB at discharge	318 (35)	205 (37)	2 [−1.3, 5.3]	104 (55)	96 (59)	4 [−3.0, 11.0]
The use of a pacifier	185 (12)	143 (11)	−1 [−3.2, 1.2]	169 (84)	124 (70)	−14 [19.9, −8.1]
The use of a nipple shield	388 (22)	286 (22)	0 [−2.9, 2.9]	52 (26)	19 (11)	−15 [20.4, −9.6]
Transferred to NICU in SSC	NR	NR	NR	68 (34)	85 (48)	14 [7.1, 20.9]

*Note.* Hospital statistics were collected for all admissions over a 1-year period. Absolute difference was calculated between the hospital care practice before and after the BFHI and Neo-BFHI designation. AD = absolute difference; NICU = neonatal intensive care unit; SSC = skin-to-skin contact; EB = exclusive breastfeeding; NA = not available; NR = not relevant.

a Exclusive breastfeeding rate included infants who received only mothers’ own milk and did not receive any medically or non-medically indicated supplementation (donated human milk). WHO standards EB rate is the sum of EB at breast after birth and medically indicated supplementation.

## Discussion

Our findings showed that implementation of the BFHI and Neo-BFHI was associated with positive changes in the HCPs’ breastfeeding attitudes as well as breastfeeding-related hospital practices. After the BFHI and Neo-BFHI implementation, improved breastfeeding attitudes were observed across each of the study units and professional groups of HCPs who participated in both data collection points. The intervention seemed to be more powerful among professions and units with lower pretest attitude scores. Previously, the BFHI principles-based education improved HCPs’ attitudes toward breastfeeding (Rosen-Carole et al., 2016; Shattnawi, 2017; Yang et al., 2018), but there are no earlier results about the association of the full implementation of BFHI on HCPs’ breastfeeding attitudes. In our study, attitudes toward breastfeeding not only became more favorable but also more consistent between units; it is important that all the hospital units are committed to the process and its goal (Esbati et al., 2019).

Breastfeeding attitudes became more facilitating and less disempowering across professional groups among HCPs who participated in both data collection points. High scores in the facilitating factor denoted that HCPs informed mothers about SSC and breastfeeding on demand and taught them to express milk by hand (Ekström et al., 2005; Ekström & Thorstensson, 2015). HCPs with higher scores in the disempowering factor indicated that professionals seemed to provide more individual counseling (Sigman-Grant & Kim, 2016). The decrease in breastfeeding antipathy indicated better breastfeeding knowledge and reduced hostile perspectives about breastfeeding (Ekström & Thorstensson, 2015). Our findings emphasize the significance of the BFHI and Neo-BFHI interventions as a basis of care, although the evidence must be confirmed in NICUs.

To ensure sufficient knowledge, competence, and skills to support breastfeeding, all HCPs received education, which also has been associated with breastfeeding attitudes (Rosen-Carole et al., 2016; Shattnawi, 2017; Yang et al., 2018), as well as the consistency of breastfeeding advice and counseling in maternity and NICU environments (Ekström & Thorstensson, 2015; Shattnawi, 2017). Our findings further support the necessity of educating and training HCPs with evidence-based breastfeeding knowledge.

Personal experiences with breastfeeding, age, and work experience were not associated with breastfeeding attitudes, contrary to what researchers have reported in previous studies (Vizzari et al., 2020; Yang et al., 2018). In this study, education was strengthened by weekly special-focus and case studies, which might have enabled professionals to reflect critically on their previous practices and their own breastfeeding experiences and change their attitudes accordingly. Reflective thinking is known to be important to professionals’ growth and development, as well as elaboration of personal assumptions, experiences, and attitudes (Bindels et al., 2018; Coward, 2018).

In addition to the improved attitudes, the implemented standards of the BFHI and Neo-BFHI improved breastfeeding-related hospital practices, for example, SSC, early breastfeeding, or supplementary nutrition (Agbozo et al., 2020; Alonso-Díaz et al., 2016; Araújo et al., 2019; Gomez-Pomar & Blubaugh, 2018; Zakarija-Grković et al., 2018; WHO, 2018). Based on these findings, BFHI interventions support evidence-based hospital practices related to breastfeeding and consistent breastfeeding counseling. This also suggests that work supporting Neo-BFHI (Nyqvist et al., 2012; 2013) breastfeeding-promoting practices is worthwhile.

Improvements in professionals’ breastfeeding attitudes were measured shortly after the BFHI and Neo-BFHI implementations; however, compliance with BFHI standards may decline rapidly after hospital designation (Zakarija-Grković et al., 2018). There is a continuous need for developing practice guidelines, monitoring, and successful training of HCPs. Self-appraisal of HCPs’ breastfeeding attitudes could be used as a follow-up measure and indicator of the status of baby-friendly practices. However, it is possible to have less favorable attitudes but still comply with a unit’s policies and breastfeeding-related practices. Causal relationships between attitudes and practices were not examined. To improve the quality of care, maintaining HCPs’ favorable attitudes toward breastfeeding is essential.

We had promising results of implementing baby-friendly practices on HCPs’ breastfeeding attitudes, though it is not possible to conclude which factors in the intervention had the greatest association with changing these attitudes. Attitudes are related to people’s feelings and emotions (Casal et al., 2017), and the success of interventions depends on the extent to which they influence emotions successfully and whether these changes in emotions influence people’s behavior (Briñol et al., 2017). Future research is needed to investigate the sustainability of achieved changes in practice. The association between professionals’ breastfeeding attitudes and the perceived advantages of breastfeeding mothers would be beneficial to investigate. Doing so could provide greater insight into the influences and working mechanisms of the BFHI and Neo-BFHI.

### Limitations

There are some limitations to note. One-group pretest-posttest design is associated with threats to internal validity (Siedlecki, 2020). We were not able to control for external events affecting the breastfeeding attitude score. All HCPs in the study units participated in this study; thus, comparison groups were not feasible. Even though the education during the intervention differed between professionals depending on previous breastfeeding education, all professionals were educated, and protocols and practices introduced. Instead of power analysis, the total population sampling method was used. Despite the 38% dropout rate, clinically significant changes in breastfeeding attitudes were achieved. Those who agreed to participate in the posttest study may have had more favorable attitudes toward breastfeeding and the practice changes required for BFHI implementation. The Breastfeeding Attitude Questionnaire (Ekström et al., 2005), used as primary outcome in this study, had poor internal consistency. Therefore, its psychometric properties require further development, especially concerning the content validity. Our results are based on data collected in a single hospital, where HCPs were motivated and the implementation process was carefully designed; thus, the results are not able to be generalizable to other settings. The cultural aspects concerning breastfeeding should always be considered.

## Conclusions

The implementation of the BFHI and Neo-BFHI interventions had significant positive association with HCPs’ breastfeeding attitudes and breastfeeding-related care practices. Generally, the professionals had more favorable attitudes toward breastfeeding after the interventions. Carefully designed and unit-based tailored implementation plans were crucial for the success of the interventions. Continuous development of practices, regular monitoring and successful training for HCPs are still needed to maintain the improved results.

## Supplemental Material

sj-docx-1-jhl-10.1177_08903344211058373 – Supplemental material for Healthcare Professionals’ Breastfeeding Attitudes and Hospital Practices During Delivery and in Neonatal Intensive Care Units: Pre and Post Implementing the Baby-Friendly Hospital Initiative Click here for additional data file. Supplemental material, sj-docx-1-jhl-10.1177_08903344211058373 for Healthcare Professionals’ Breastfeeding Attitudes and Hospital Practices During Delivery and in Neonatal Intensive Care Units: Pre and Post Implementing the Baby-Friendly Hospital Initiative by Heli Mäkelä, Anna Axelin, Terhi Kolari, Tuula Kuivalainen and Hannakaisa Niela-Vilén in Journal of Human Lactation
