# Supplementary material for: Healthcare Professionals’ Breastfeeding Attitudes and Hospital Practices During Delivery and in Neonatal Intensive Care Units: Pre and Post Implementing the Baby-Friendly Hospital Initiative
Source: J Hum Lact. 2021 Nov 28;38(3):537–47. doi: 10.1177/08903344211058373 (PMC9329761; doi:10.1177/08903344211058373)
Supplement: sj-docx-1-jhl-10.1177_08903344211058373 – Supplemental material for Healthcare Professionals’ Breastfeeding Attitudes and Hospital Practices During Delivery and in Neonatal Intensive Care Units: Pre and Post Implementing the Baby-Friendly Hospital Initiative [file sj-docx-1-jhl-10.1177_08903344211058373.docx]

#
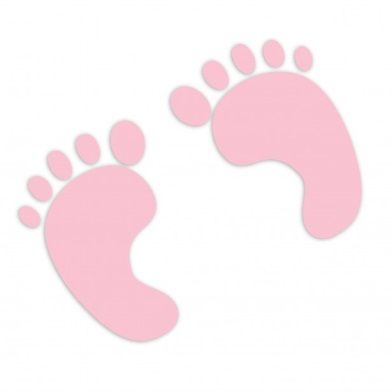
Vauvamyönteisyysohjelman vaikutus imetykseen,

# imetysohjaukseen ja imetysasenteisiin

_______________________________________________________

*Kyselylomake henkilökunnalle*

Taustatiedot

1. Nimesi _____________________________________ (*tietoa käytetään seurantakyselyn kohdentamisessa; nimesi tulee vain tutkijoiden tietoon*)
2. Ikäsi ______ vuotta
3. Sukupuolesi: 1 Nainen

2 Mies

1. Korkein ammatillinen koulutuksesi/tutkintosi ______________________________
2. Ammattisi ___________________________________
3. Millä osastolla pääasiallisesti työskentelet? _________________________
4. Kuinka pitkään olet työskennellyt nykyisellä osastollasi? _________ vuotta
5. Kuinka pitkään olet työskennellyt nykyisessä ammatissasi? _________ vuotta
6. Onko sinulla jotakin imetysohjauskoulutusta?

1 Kyllä

2 Ei

1. Mikäli vastasit Kyllä, kuvaa mikä ja miten laaja koulutus Sinulla on ja minä vuonna olet koulutukseen osallistunut

_____________________________________________________________________

_____________________________________________________________________

1. Koetko tarvitsevasi lisää imetyskoulutusta

1 Kyllä

2 En

1. Onko Sinua itseäsi imetetty?

1 Kyllä

2 Ei

3 En tiedä

1. Oletko itse / onko kumppanisi imettänyt?

1 Kyllä

2 En

3 Minulla ei ole lapsia

1. Mikäli vastasit Kyllä, kuvaa kauanko olet imettänyt (kunkin lapsen kohdalla erikseen)

_____________________________________________________________________

_____________________________________________________________________

1. Miten tärkeäksi koet imetyksen?

Ei lainkaan tärkeä 1 2 3 4 5 6 7 8 9 10 Erittäin tärkeä
